# Comparison of CNN Algorithms on Hyperspectral Image Classification in Agricultural Lands

**DOI:** 10.3390/s20061734

**Published:** 2020-03-20

**Authors:** Tien-Heng Hsieh, Jean-Fu Kiang

**Affiliations:** Graduate Institute of Communication Engineering, National Taiwan University, Taipei 10617, Taiwan; R04942090@ntu.edu.tw

**Keywords:** convolutional neural network (CNN), hyperspectral image (HSI), agriculture, principal component analysis (PCA)

## Abstract

Several versions of convolutional neural network (CNN) were developed to classify hyperspectral images (HSIs) of agricultural lands, including 1D-CNN with pixelwise spectral data, 1D-CNN with selected bands, 1D-CNN with spectral-spatial features and 2D-CNN with principal components. The HSI data of a crop agriculture in Salinas Valley and a mixed vegetation agriculture in Indian Pines were used to compare the performance of these CNN algorithms. The highest overall accuracy on these two cases are 99.8% and 98.1%, respectively, achieved by applying 1D-CNN with augmented input vectors, which contain both spectral and spatial features embedded in the HSI data.

## 1. Introduction

Hyperspectral images (HSIs) contain abundant spectral-spatial information for versatile applications in agriculture, mineralogy, surveillance, physics, astronomy, chemical imaging and environmental sciences [1]. Classifications based on HSIs face the challenges of redundant features, limited number of available training samples and high dimensionality of data [2]. Many methods have been applied to classify HSIs, including random forest (RF) [3], *k*-nearest neighbors [4], multinomial logistic regression (MLR) [5], support vector machine (SVM) [6], convolutional neural network (CNN) [7], deep belief network (DBN) [8] and stacked auto-encoder (SAE) [9]. Among these methods, deep CNN has the potential of learning high-level spatial and spectral features [10,11].

Feature extraction and feature selection approaches were proposed to curtail the redundancy of information among hyperspectral bands [12]. By feature extraction, a projection matrix is used to map the original spectral data to a feature space while holding the dominant spectral information [13]. Typical feature extraction algorithms include principal component analysis (PCA) [14], linear discriminant analysis (LDA) [15], manifold learning [16], nonnegative matrix factorization (NMF) [17] and spatial-spectral feature extraction [18].

Feature selection means selecting part of the original bands based on proper criterion [19]. Typical feature selection algorithms include multitask sparsity pursuit [20], structure-aware [21], support vector machine [22], hypergraph model [23], sparse Hilbert–Schmidt independence criterion [24] and nonhomogeneous hidden Markov chain model [25]. Different measures were used to select preferred bands, including mutual information [12], information divergence [13], variance [26] and local spatial information [27]. However, these algorithms are time-consuming because the classifiers must be trained and tested again as the set of selected bands is changed. Pixels in an HSI are usually spatially correlated to their adjacent pixels [28], which can be exploited to complement the spectral information to achieve higher classification rate [29]. In [30], a spectral-spatial semisupervised training set construction was proposed to mitigate the problem of labeled data scarcity, in which unlabeled pixels are recruited for a class training subset if they belong to the same spectral cluster and are in the spatial neighborhood of a labeled pixel.

Deep spectral features embedded in the HSIs of agricultural vegetation can be physically related to the photosynthetic pigment absorption in the wavelengths of 400–700 nm, large spectral slope in 700–750 nm [31,32], liquid water inflection point in 1080–1170 nm [33], absorption by various leaf waxes and oils in 1700–1780 nm and cellulose absorption around 2100 nm, to name a few. The spectral features relevant to soil properties mainly appear in 2100–2300 nm [34,35]. These spectral features can be exploited for applications like precision agriculture [36,37], noxious weed mapping for rangeland management [38,39], forest health monitoring [40,41], vegetation stress analysis [42,43] and carbon sequestration site monitoring [44].

CNN has the potential of exploiting deep-level features embedded in its input data for classification, making it suitable for terrain classification with HSI data that contain both spatial and spectral features. Although CNNs have been widely used for classification in agricultural lands, there are always some outliers or misclassification between similar classes which contain similar spatial and spectral features. In this work, we present several versions of CNN, each taking different types of input vectors that include more feature information, to resolve these issues. These CNNs were trained and tested on the HSIs of Salinas Valley and Indian Pines, respectively. The former is a crop agriculture, the latter contains two-thirds crop agriculture and one-third forest or other natural perennial vegetation. The rest of this article is organized as follows. The 1D-CNN with input vector composed of pixelwise spectral data and spectral-spatial data are presented in Section 2 and Section 3, respectively, The 2D-CNN with input layer of principal components is presented in Section 4, simulation results are presented and analyzed in Section 5, and some conclusions are drawn in Section 6.

## 2. 1D-CNN with Pixelwise Spectral Data

**Figure 1:**
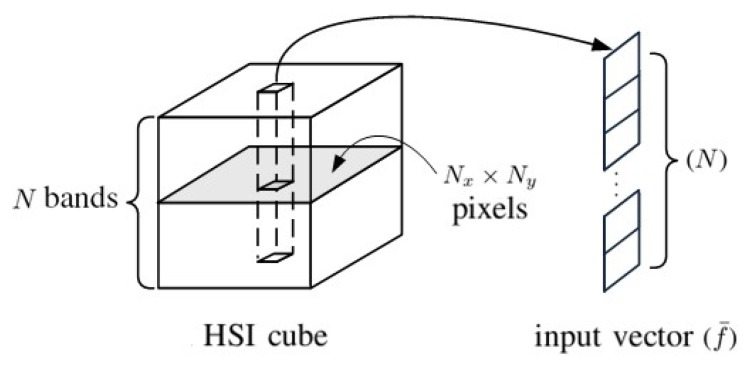
The 1D input vector retrieved from a hyperspectral image (HSI) cube.

**Figure 2:**
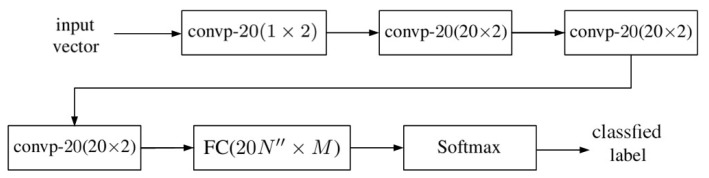
Schematic of a 1D convolutional neural network (CNN).

Figure 1 shows an HSI cube which is composed of $N_{x}\times N_{y}$ pixels and each pixel contains spectral data in *N* bands. A one-dimensional (1D) input vector is prepared for each pixel by extracting the spectral data from that pixel. The input vectors from a selected set of training pixels are used to train the 1D-CNN shown in Figure 2, then the input vectors from another set of testing pixels are used to evaluate the performance of the 1D-CNN.

**Figure 3:**
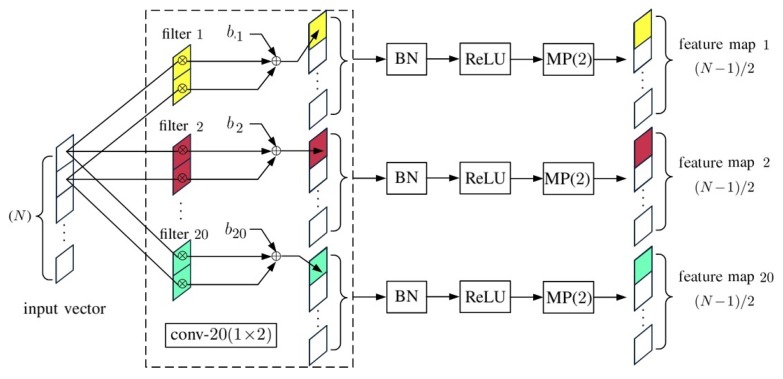
Schematic of convp-20(1×2) layer.

**Figure 4:**
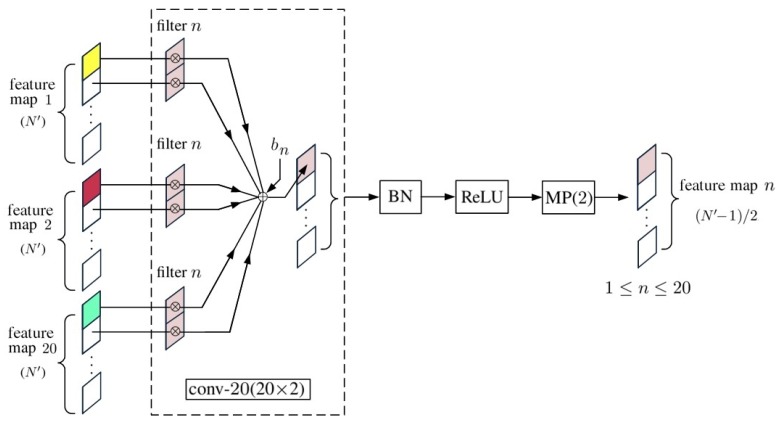
Schematic of convp-20(20×2).

**Figure 5:**
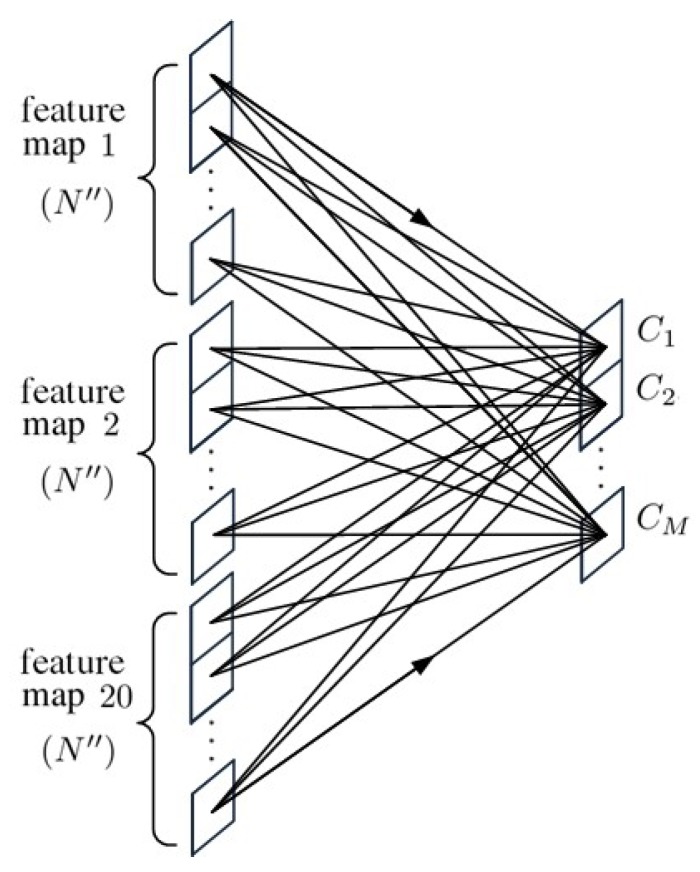
Schematic of FC$\left(20N^{\prime \prime }\times M\right)$.

In the schematic of a 1D-CNN shown in Figure 2, the compositions of convp-n(1×2) and convp-20(20×2) are shown in Figure 3 and Figure 4, respectively, and FC$\left(20N^{\prime \prime }\times M\right)$ is a fully connected layer shown in Figure 5.

Figure 3 shows the schematic of a convp-20(1×2) layer, where conv-n(1×2) is a convolutional layer composed of *n* filters of kernel size two, taking one input vector. The outputs of conv-n(1×2) are processed with batch normalization (BN), rectified linear unit (ReLU) activation function and maxpooling, MP(2), in sequence. The BN is used to make the learning process less sensitive to initialization. The input to a BN is a mini-batch of $M^{\prime }$ input vectors, ${\bar{x}}_{m}={\left[x_{m1},x_{m2},\cdots ,x_{mN}\right]}^{t}$ with $1\leq m\leq M^{\prime }$. The mean value and variance of the *n*th band in the *b*th mini-batch are computed as [45]

(1) $$\begin{array}{c} \mu_{bn}=\frac{1}{M^{\prime }}\sum_{m=1}^{M^{\prime }}x_{mn},\;\;\sigma_{bn}^{2}=\frac{1}{M^{\prime }}\sum_{m=1}^{M^{\prime }}{\left(x_{mn}-\mu_{bn}\right)}^{2} \end{array}$$

Then, the original input vectors in the *b*th mini-batch are normalized as

(2) $$\begin{array}{c} {\tilde{x}}_{mn}=\frac{x_{mn}-\mu_{bn}}{\sqrt{\sigma_{bn}^{2}+\epsilon }} \end{array}$$

where ϵ is a regularization constant to avoid divergence when $\sigma_{bn}^{2}$ is too small. To further increase the degree-of-freedom in the subsequent convolutional layers, the normalized input vectors are scaled and shifted as

(3) $$\begin{array}{c} y_{mn}=\gamma_{n}{\tilde{x}}_{mn}+\beta_{n} \end{array}$$

where the offset $\beta_{n}$ and the scaling factor $\gamma_{n}$ are updated during the training phase. The ReLU activation function is defined as y=max{0,x}, with input *x* and output *y*. The maxpooling function, MP(*ℓ*), reduces the computational load by picking the maximum from *ℓ* input data, which preserves the main characteristics of feature maps at the cost of coarser resolution.

Figure 4 shows the composition of a convp-20(20×2) layer, where conv-n(20×2) is a convolutional layer composed of *n* filters of kernel size two, taking 20 input feature maps.

Figure 5 shows the schematic of a fully connected layer, FC$\left(20N^{\prime \prime }\times M\right)$, which connects the feature maps from the last convolutional layer, convp-20(20×2), to the input of the softmax function for final classification. The softmax function is a normalized exponential function defined as

(4) $$\begin{array}{c} \mathrm{softmax}\left(\bar{x}\right)={\left(\sum_{m=1}^{M}e^{x_{m}}\right)}^{-1}e^{\bar{x}} \end{array}$$

which enhances the largest component in $\bar{x}$ while making the sum of all its output components equal to one.

### Band Selection Approach

**Figure 6:**
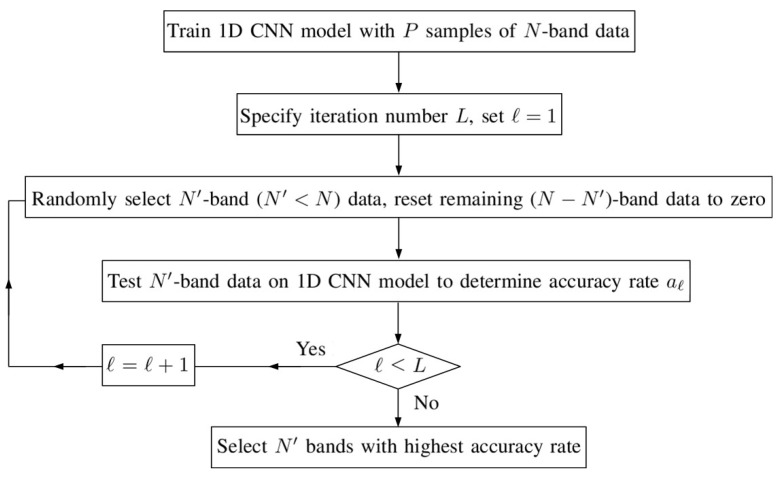
Flowchart of band selection (BS) approach based on CNN (BSCNN).

Figure 6 shows the flowchart of a band selection (BS) approach based on CNN (BSCNN), which selects the best combination of spectral bands for classification. A CNN was first trained by using all the *N* spectral bands of training pixels, and its configuration remained the same during the subsequent band selection process. The BS process was executed *L* times. In each time, $N^{\prime }$ bands $\left(N^{\prime }<N\right)$ were randomly selected and the data in the other $\left(N-N^{\prime }\right)$ bands were reset to zero in the input vector. Among all these *L* combinations, the $N^{\prime }$ bands delivering the highest overall accuracy is adopted for retraining the CNN for classification.

**Figure 7:**
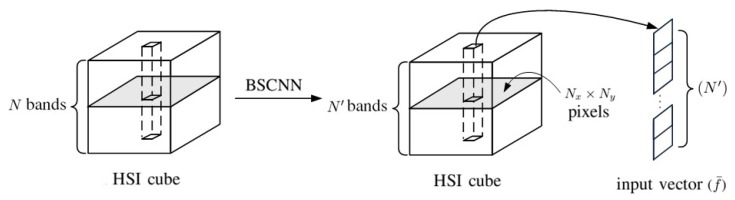
Input vectors to the 1D-BSCNN.

Figure 7 shows the preparation of input vectors with the selected $N^{\prime }$ bands to retrain the 1D-CNN, as configured in Figure 2. The newly trained 1D-CNN is be used for classification.

## 3. 1D-CNN with Spectral-Spatial Data

**Figure 8:**
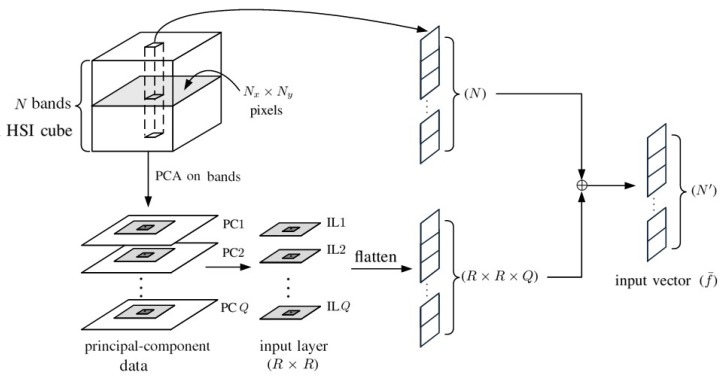
Preparation of an augmented input vector by concatenating pixelwise spectral bands and the principal component analysis (PCA) data surrounding the target pixel.

Figure 8 shows the preparation of an augmented input vector by concatenating the spectral bands of the target pixel with the PCA data surrounding the target pixel, exploiting the spatial correlation between neighboring pixels. The PCA is first applied to all the spectral bands of each pixel to extract the first *Q* principal components. Then, the first *Q* principal components of all the R×R pixels surrounding the target pixel are collected, vectorized and concatenated to the original *N* bands of the target pixel to form an augmented input vector of dimension N+R×R×Q, to be input to the 1D-CNN.

## 4. 2D-CNN with Principal Components

**Figure 9:**
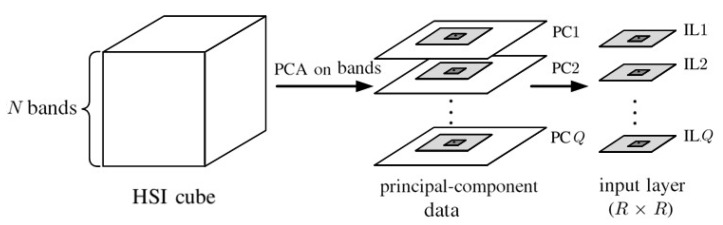
Preparation of input layers for 2D-CNN.

**Figure 10:**
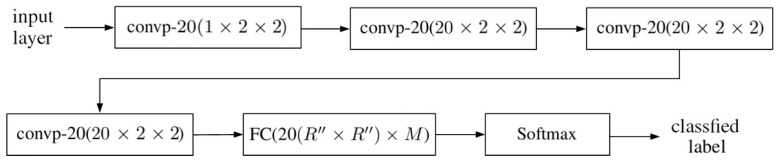
Schematic of the 2D-CNN.

Figure 9 shows the preparation of input layers, composed of principal components from each pixel, to be input to the 2D-CNN shown in Figure 10. The PCA is first applied to all the *N* spectral bands of each pixel to extract the first *Q* principal components [46]. The *Q* principal components from each of the R×R pixels surrounding the target pixel form an input layer associated with the target pixel. The PCA extracts the main features in the spectral dimension and exploits the spatial features embedded in the hyperspectral data.

**Figure 11:**
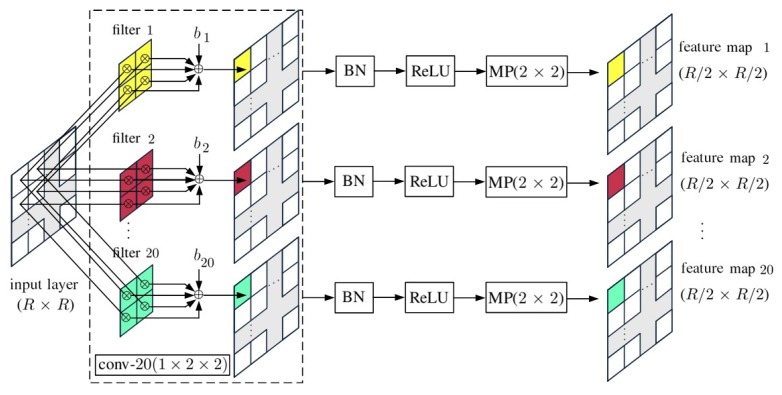
Schematic of convp-20(1×2×2).

**Figure 12:**
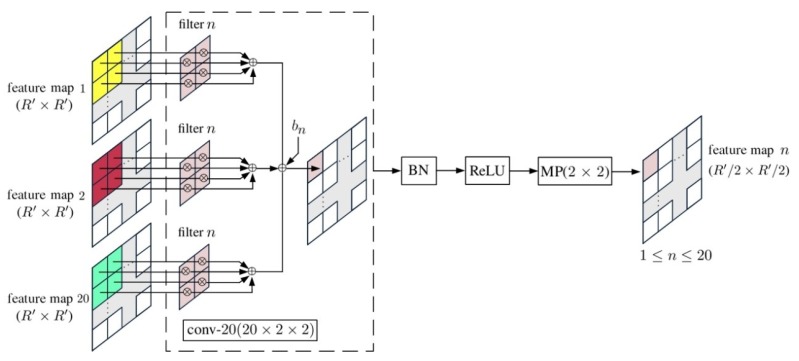
Schematic of convp-20(20×2×2).

**Figure 13:**
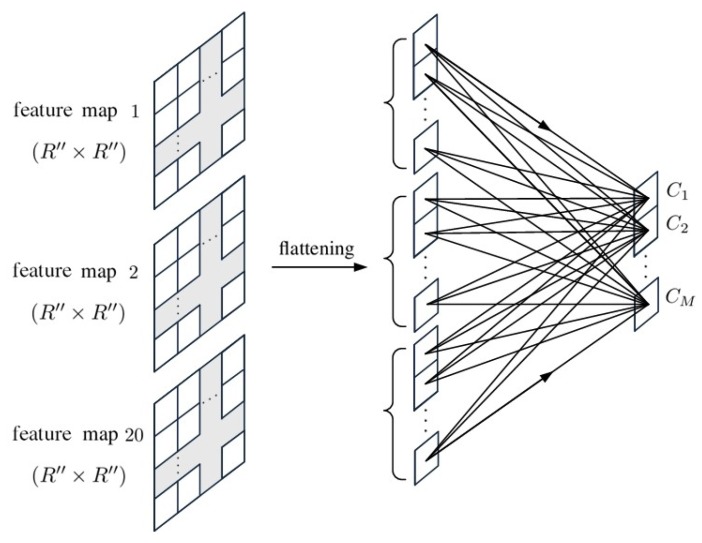
Schematic of FC$\left(20\left(R^{\prime \prime }\times R^{\prime \prime }\right)\times M\right)$.

Figure 10 shows the schematic of the 2D-CNN used in this work, where the compositions of convp-n(1×2×2) and convp-20(20×2×2) are shown in Figure 11 and Figure 12, respectively, FC$\left(20R^{\prime \prime }\times R^{\prime \prime }\times M\right)$ is a fully connected layer shown in Figure 13. Cascading four convp-20(1×2×2) layers makes the resulting 2D-CNN highly nonlinear and enables it to recognize more abstract spatial-spectral features embedded in the hyperspectral data.

Figure 11 shows the schematic of a convp-20(1×2×2) layer, where conv-n(1×2×2) is a convolutional layer composed of *n* filters of kernel size 2×2, taking one input layer. The outputs of conv-n(1×2×2) are processed with BN, ReLU activation function and MP(2×2) in sequence.

Figure 12 shows the composition of a convp-20(20×2×2) layer, where conv-n(20×2×2) is a convolutional layer composed of *n* filters of kernel size 2×2, taking 20 input feature maps.

Figure 13 shows the composition of a fully connected layer, FC$\left(20\left(R^{\prime \prime }\times R^{\prime \prime }\right)\times M\right)$, which connects the feature maps from the last convolutional layer, convp-20(20×2×2), to the input of softmax function for final classification.

## 5. Simulations and Discussions

### 5.1. Salinas Valley HSI

**Figure 14:**
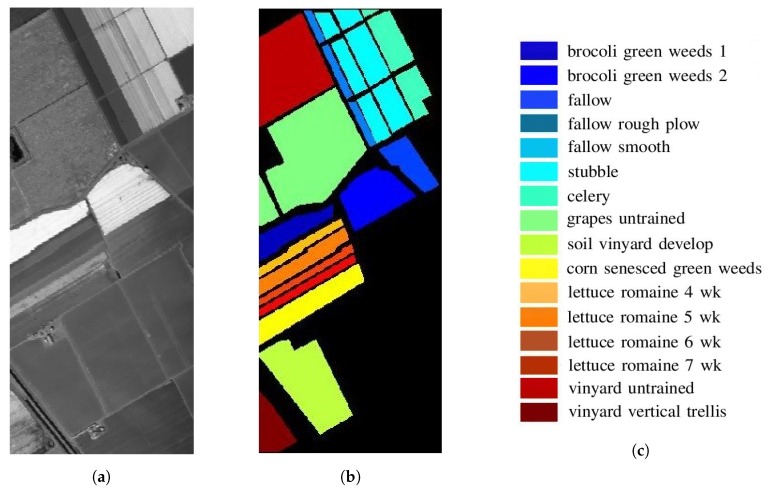
Images of Salinas Valley, centered at $36^{{}^{\circ}}40^{\prime }39.8568^{\prime \prime }$ N, $121^{{}^{\circ}}39^{\prime }19.8072^{\prime \prime }$ W [47], area size is 1895 × 803 m, (**a**) grey-tone image, (**b**) ground truth, (**c**) legends of 16 classes.

Figure 14 shows the image and ground truth, respectively, in Salinas Valley, which were acquired with the Airborne Visible/Infrared Imaging Spectrometer (AVIRIS) sensor in October 1998 [47]. The HSI is composed of 512×217 pixels, with spatial resolution of 3.7 m. The spectral data in wavelengths of 400–2500 nm were recorded in 224 bands, among which bands 108–112, 154–167 and 224 are removed for the concern of dense water vapor and atmospheric effects, leaving 204 more reliable bands. Table 1 lists the ground truth of 54,129 pixels in 16 classes [47].

The main objective of the AVIRIS project was to identify, measure and monitor the composition of Earth surface and atmosphere, based on the signatures of molecular absorption and particle scattering. Research with AVIRIS data were focused on understanding the processes related to global environment and climate change [48].

In the training phase of this work, 50% of pixels were randomly selected, labeled by ground-truth data, to determine the weights and biases associated with each neuron. Mini-batches of size $M^{\prime }=16$ were used over 200 training epochs. The other 50% of pixels were then used in the testing phase to evaluate the classification performance.

**Figure 15:**
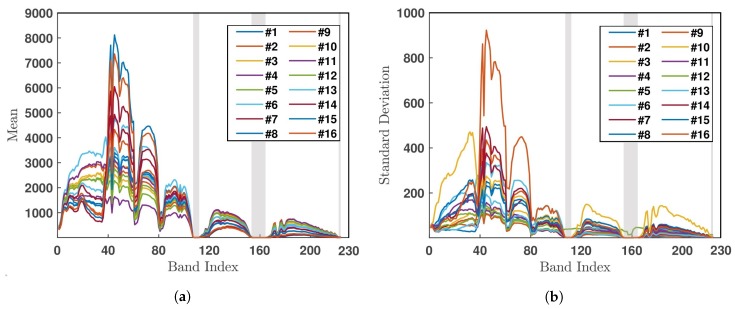
(**a**) Mean value and (**b**) standard deviation of all the pixels in each of the 16 classes, over all 224 bands in Salinas Valley HSI.

Figure 15 shows the mean value and standard deviation of all the pixels in each of the 16 classes, over all 224 bands. The bands of dense water vapor and atmospheric effects are marked by a grey shade.

**Figure 16:**
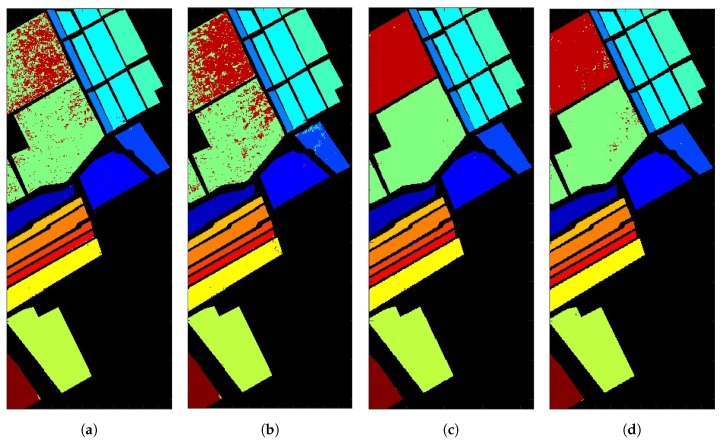
Classification images on Salinas Valley HSI, (**a**) 1D-CNN with 204 bands, overall accuracy (OA) is 91.8%, (**b**) BSCNN with 70 selected bands, OA is 93.2%, (**c**) 1D-CNN with augmented input vectors of 645 (21×21+204) bands, OA is 99.8%, (**d**) 2D-CNN with input layer composed of one principal component from each pixel, OA is 99%, color code the same as in Figure 14.

Figure 16a shows the classification image with 1D-CNN applied to 204 selected bands. The overall accuracy (OA) is 91.8%, which is the ratio of pixels correctly classified and the total number of testing pixels. Table 2a lists the producer accuracy (PA) of each class, which is the ratio of pixels correctly classified to a specific class and the total number of pixels which are classified to that specific class. The PAs of classes #8 and #15 are 91.9% and 54.8%, respectively, consistent with the observation on Figure 16a that classes #8 and #15 are apparently misclassified. Also notice that some spectral curves in Figure 15 nearly overlap in certain bands, which may cause errors in classification.

**Figure 17:**
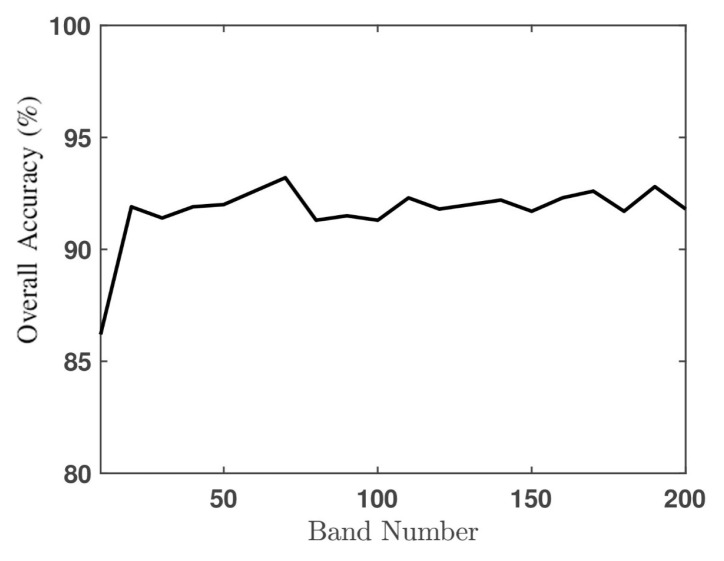
Effect of band number in BSCNN on overall accuracy.

Figure 17 shows the effect of band number on the overall accuracy, which indicates that choosing $N^{\prime }=70$ bands renders the highest overall accuracy. Table 2b lists the PAs of all 16 classes on the Salinas Valley HSI by applying BSCNN with 70 bands. By comparing with the results in Table 2a, the PA on class #8 decreases from 91.9% to 86.1% and that on class #15 increases from 54.8% to 74.8%. Figure 16b shows the classification image by applying the BSCNN.

Figure 8 shows the preparation of an augmented input vector by concatenating the *N* spectral bands of the target pixel and *Q* principal components from each of the R×R pixels surrounding the target pixel. By choosing N=204, Q=1 and R=21, the augmented input vector has the dimension of $N^{\prime }=645$. With additional spatial information, the accuracy rate is expected to improve [49]. Figure 16c shows the classification image with augmented input vectors of 645 bands. Table 2c lists the PAs of all 16 classes, and the overall accuracy is 99.8%. By comparing Table 2a with Table 2b, the PAs on classes #8 and #15 are significantly increased to 99.8% and 99.7%, respectively.

Figure 16d shows the classification image by using 2D-CNN, with one principal component from each pixel to form an input layer. Table 2d lists the PAs of all 16 classes, and the overall accuracy is 99%.

In summary, the OA of 1D-CNN with 204 selected bands is the lowest of 91.8%, that of BSCNN with 70 selected bands is 93.2%, that of 1D-CNN with augmented input vectors of 645 bands is the highest of 99.8% and that of 2D-CNN with one principal component from each pixel is 99%.

### 5.2. Indian Pines HSI

**Figure 18:**
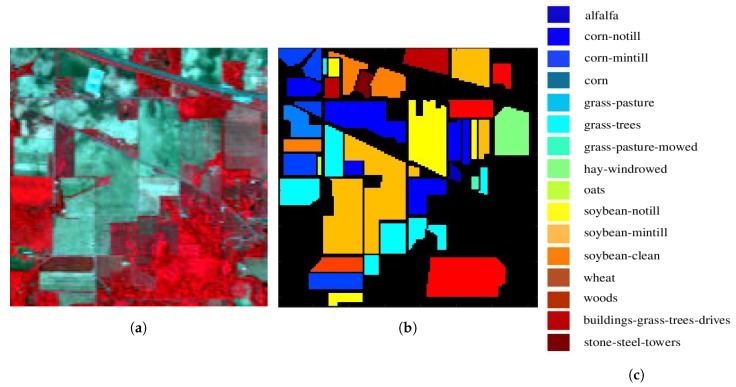
Images of Indian Pines, centered at $40^{{}^{\circ}}27^{\prime }37.9^{\prime \prime }$ N, $87^{{}^{\circ}}00^{\prime }32.7^{\prime \prime }$ W [50], area size is 3.2 × 3.2 km, (**a**) false color image, (**b**) ground truth, black area is unlabeled, (**c**) legends of 16 classes.

Figure 18 shows a testing site in the Indian Pines, which was recorded on 12 June 1992, with AVIRIS sensors over the Purdue University Agronomy farm, northwest of West Lafayette. The image is composed of 145×145 pixels, each containing 220 spectral reflectance bands in wavelengths of 400–2500 nm. The number of bands is reduced to 200 after removing bands 104–108, 150–163 and 220, which suffer significant water absorption. Two-thirds of the test site was covered with agriculture land and one-third with forest or other natural perennial vegetation. There were also two dual-lane highways, a rail line, some low-density housings, other man-made structures and local roads. At the time of recording, some crops were growing, corn and soybeans were in their early stage of growth, with less than 5% coverage. Table 3 lists the available ground truth in 16 classes, which are not mutually exclusive.

In the training phase of this work, 50% of pixels were randomly selected, labeled by ground-truth data, to determine the weights and biases associated with each neuron. Mini-batches of size $M^{\prime }=16$ were used over 200 training epochs. The other 50% of pixels were then used in the testing phase to evaluate the classification performance.

**Figure 19:**
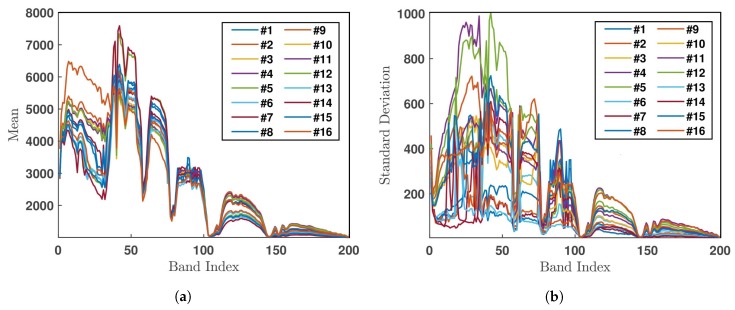
(**a**) Mean value and (**b**) standard deviation of all the pixels in each of 16 classes over all 200 bands in Indian Pines HSI.

Figure 19 shows the mean value and standard deviation of all the pixels in each of the 16 classes, over the 200 selected bands.

**Figure 20:**
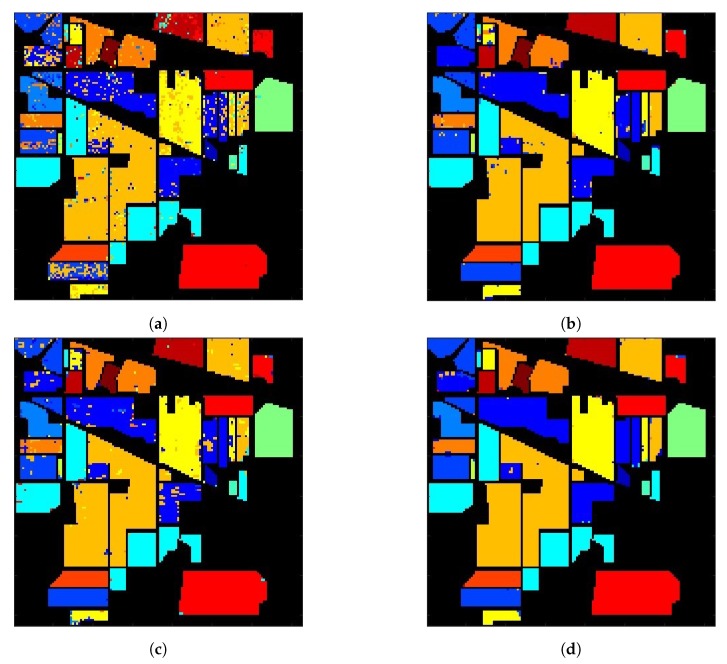
Classification images on Indian Pines HSI: (**a**) 1D-CNN with 200 bands, OA is 83.4%, (**b**) 1D-CNN with augmented input vectors of 641 (21×21+200) bands, OA is 95.4%, (**c**) 2D-CNN with input layer composed of one principal component from each pixel, OA is 91.5%, (**d**) 1D-CNN with augmented input vectors of 1964 ((21×21)×4+200) bands, OA is 98.1%, color code the same as in Figure 18.

Figure 20a shows the classification image with 1D-CNN applied to 200 selected bands, and the overall accuracy is 83.4%. Table 4a lists the PAs of all 16 classes, in which the lowest PAs are 55.2%, 57.1% and 62.2%, on classes #3, #7 and #15, respectively.

Figure 20b shows the classification image with augmented input vectors of 641 bands, where we choose N=200, Q=1 and R=21. Table 4b lists the PAs of all 16 classes, with an overall accuracy of 95.4%. Compared to Table 4a, the PAs on classes #3, #7 and #15 are improved to 94%, 94.7% and 99.5%, respectively.

Figure 20c shows the classification images by applying 2D-CNN, with the input layer composed of one principal component from each pixel. Table 4c lists the PAs of all 16 classes, with the overall accuracy of 91.5%.

In summary, the OA of 1D-CNN with 200 selected bands is the lowest of 83.4%, that of 1D-CNN with augmented input vectors of 641 bands is 95.4%, and that of 2D-CNN with one principal component from each pixel is 91.5%.

**Figure 21:**
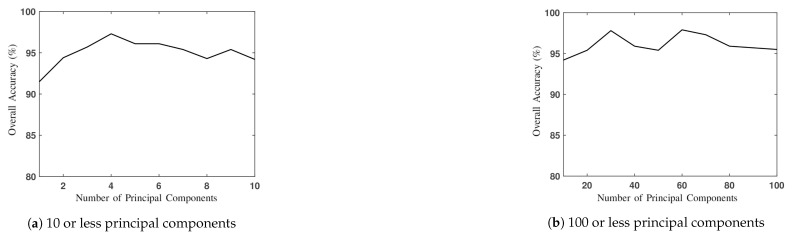
Overall accuracy versus number of principal components in 2D-CNN.

Figure 21 shows the overall accuracy of 2D-CNN with different numbers of principal components. The highest OA is slightly below 98%, with 4, 30 or 60 principal components. Figure 20d shows the classification image by applying 1D-CNN with augmented input vectors of 1964 bands, where we choose N=200, Q=4 and R=21. Table 4d lists the PAs of all 16 classes, with the overall accuracy of 98.1%. Compared to Table 4a, the PAs on classes #3, #7 and #15 are significantly improved to 98.6%, 100% and 99.5%, respectively.

The computational time for training and testing these CNNs as well as the resulting accuracy were affected by the size of input vector, input layer, convolution kernel, batch and epoch. Table 5 lists the CPU time for each CNN developed in this work, on a desktop PC with Intel^®^ Core™ i7-8700 processor 3.2 GHz.

Both sets of HSI data used in this work were recorded in about 200 bands and classified into 16 labels. The pixel numbers are 54,129 and 21,025, respectively. The complexity of the CNNs adopted in this work seems suitable to these HSI datasets. It is conjectured that more complicated CNN configuration should be considered if more bands or more labels are involved. The results on these two cases show that the overall accuracy of the 1D-CNN with augmented input vectors is higher than 1D-CNN, BSCNN and 2D-CNN. The results of 2D-CNN turn out to be more accurate than conventional 1D-CNN, indicating that the spatial feature embedded in the spectral data can be useful. A small percentage of misclassification between similar classes can be resolved by applying the 1D-CNN with augmented input vectors which contain both the spatial and spectral features embedded in the HSI data.

## 6. Conclusions

Both the spectral and spatial features of HSI are exploited to increase the overall accuracy of image classification with several versions of 1D-CNN and 2D-CNN. The PCA was applied to extract significant spectral information while reducing the data dimension. These CNNs were applied to the HSI data on Salinas Valley and Indian Pines, respectively, to compare their accuracy rates of classification. The selection of band number and principal components was investigated by simulations. The highest OA on the Salinas Valley HSI is 99.8%, achieved by applying 1D-CNN to augmented input vectors of 645 bands, with one principal component from 21 × 21 pixels surrounding the target pixel. The highest OA on the Indian Pines HSI is 98.1%, achieved by applying 1D-CNN to augmented input vectors of 1964 bands, with four principal components from 21 × 21 pixels surrounding the target pixel. Possible misclassification between similar labels can be resolved by augmenting the input vectors to include more spatial and spectral features embedded in the HSI data.

## Figures and Tables

**Table 1:** Summary of ground truth in Figure 14.

#	Class	Sample Number
1	broccoli green weeds 1	2009
2	broccoli green weeds 2	3726
3	fallow	1976
4	fallow rough plow	1394
5	fallow smooth	2678
6	stubble	3959
7	celery	3579
8	grapes untrained	11,271
9	soil vineyard develop	6203
10	corn senesced green weeds	3278
11	lettuce romaine 4 weeks	1068
12	lettuce romaine 5 weeks	1927
13	lettuce romaine 6 weeks	916
14	lettuce romaine 7 weeks	1070
15	vineyard untrained	7268
16	vineyard vertical trellis	1807

**Table 2:** Producer accuracy (%) on Salinas Valley HSI: (a): 1D-CNN with 204 bands, (b): BSCNN with 70 selected bands, (c): 1D-CNN with augmented input vectors of 645 bands, (d): 2D-CNN with input layer composed of one principal component from each pixel.

	#1	#2	#3	#4	#5	#6	#7	#8	#9	#10	#11	#12	#13	#14	#15	#16	OA
(a)	99.8	99.8	99.8	99.9	98.1	100	99.8	**91.9**	99.6	98.2	92	99.8	100	97.5	**54.8**	99.7	91.8
(b)	99.8	99.8	95.6	99.4	98.3	99.9	99.8	**86.1**	100	97.5	99.3	100	99.1	99.1	**74.8**	99.3	93.2
(c)	99.8	99.9	100	99.9	99.9	100	99.9	**99.8**	99.9	99.7	100	99.9	99.8	100	**99.7**	99.9	99.8
(d)	99.7	100	99.8	100	99.6	100	100	97.6	99.7	99.9	100	100	100	100	97.1	99.8	99

**Table 3:** Summary of ground truth in Figure 18 [50].

#	Class	Sample Number
1	alfalfa	46
2	corn-notill	1428
3	corn-mintill	830
4	corn	237
5	grass-pasture	483
6	grass-trees	730
7	grass-pasture-mowed	28
8	hay-windrowed	478
9	oats	20
10	soybean-notill	972
11	soybean-mintill	2455
12	soybean-clean	593
13	wheat	205
14	woods	1265
15	buildings-grass-trees-drives	386
16	stone-steel-towers	93

**Table 4:** Producer accuracy (%) on Indian Pines HSI: (a): 1D-CNN with 200 bands, (b): 1D-CNN with augmented input vectors of 641 bands, (c): 2D-CNN with input layer composed of one principal component from each pixel, (d): 1D-CNN with augmented input vectors of 1964 bands.

	#1	#2	#3	#4	#5	#6	#7	#8	#9	#10	#11	#12	#13	#14	#15	#16	OA
(a)	83.3	76.1	**55.2**	82	94.4	96.5	**57.1**	98.7	77.8	73.3	87.2	84.9	99	96.6	**62.2**	91.7	83.4
(b)	95.2	92.8	**94**	97.5	89.1	99.2	**94.7**	99.6	71.4	89.7	97.5	88.4	100	98.9	**99.5**	100	95.4
(c)	90.5	82.2	**87.4**	92	90.7	96.5	**100**	100	58.3	82.1	95.1	88	100	97.7	**96.9**	97.7	91.5
(d)	100	94.9	**98.6**	97.5	99.2	99.7	**100**	100	90.9	96.7	98.8	97.2	99	99.2	**99.5**	98.2	98.1

**Table 5:** Computational time for training and testing CNNs.

HSI on Salinas Valley	CPU Time	HSI on Indian Pines	CPU Time
1D-CNN with 204 bands	1 h 43 min	1D-CNN with 200 bands	18 min
BSCNN with 70 selected bands	1 h 35 min	1D-CNN with augmented input vectors of 641 bands	26 min
1D-CNN with augmented input vectors of 645 bands	6 h 58 min	2D-CNN with input layer composed of one principal component from each pixel	17 min
2D-CNN with input layer composed of one principal component from each pixel	1 h 32 min	1D-CNN with augmented input vectors of 1964 bands	1 h 27 min
